# Supplementary material for: Phylotypic Profiling, Distribution of Pathogenicity Island Markers, and Antimicrobial Susceptibility of Escherichia coli Isolated from Retail Chicken Meat and Humans
Source: Antibiotics (Basel). 2022 Sep 4;11(9):1197. doi: 10.3390/antibiotics11091197 (PMC9495032; doi:10.3390/antibiotics11091197)
Supplement: Supplementary file 1 [file antibiotics-11-01197-s001.zip › antibiotics-1847285-supplementary.pdf]

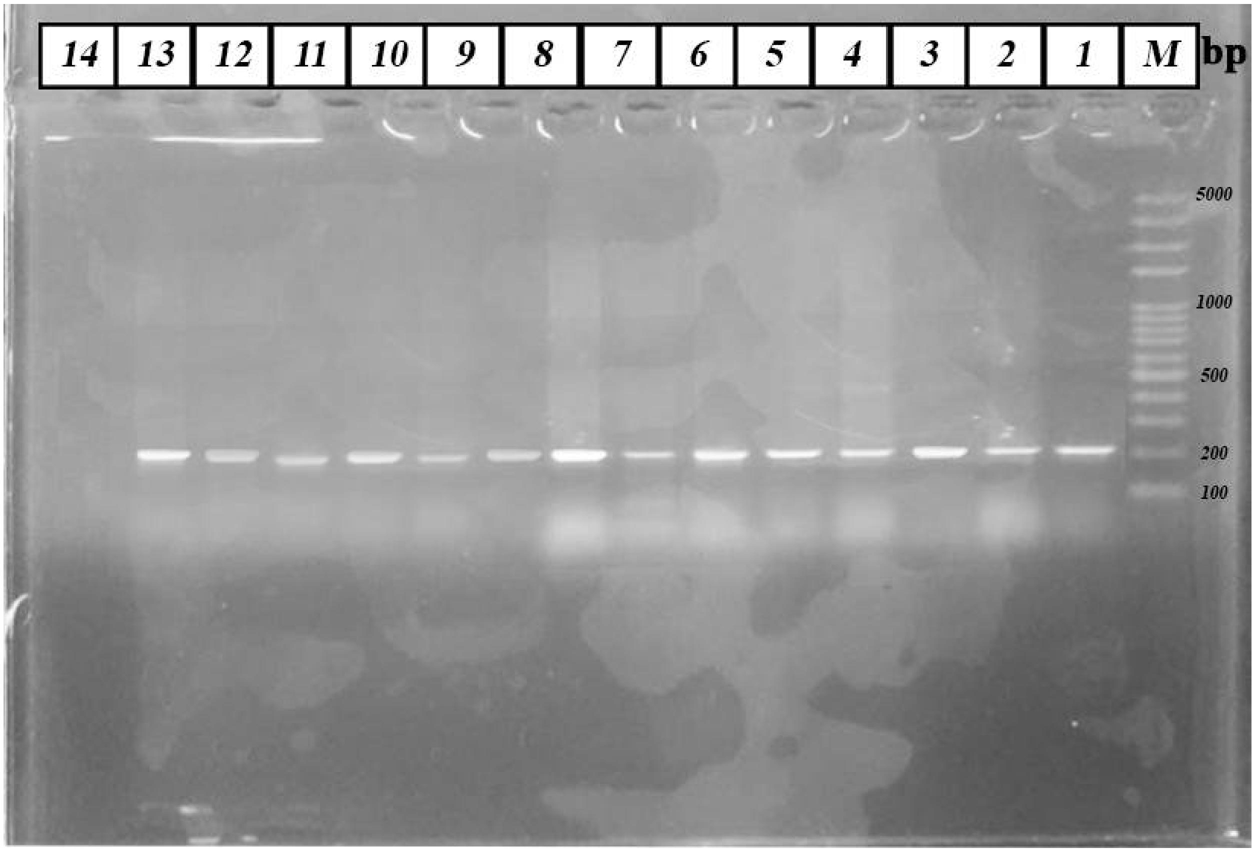

Figure S1: Agarose gel electrophoresis showing amplification of 16S small subunit rRNA gene of E. coli at 200 bp

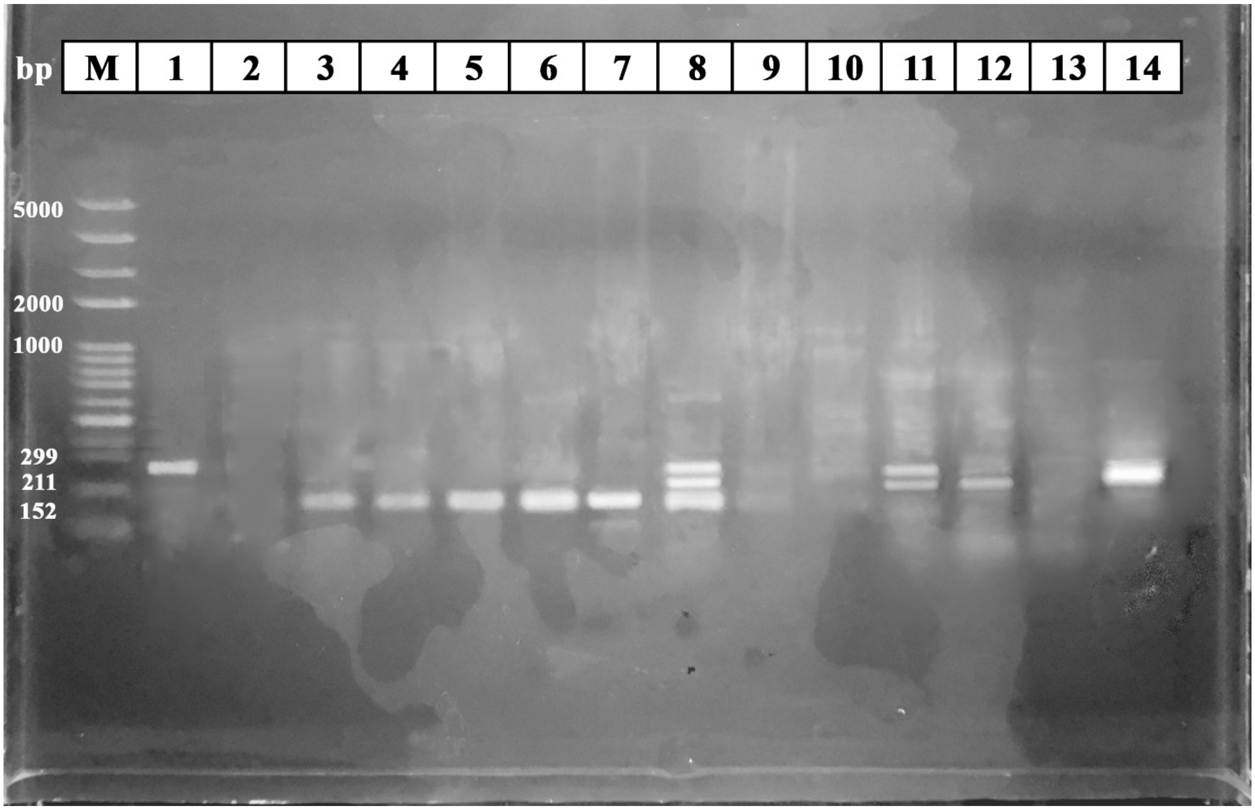

Figure S2: Agarose gel electrophoresis showing ECOR phylogenetic grouping of representative E. coli isolates according to PCR: lane M: 100 bp DNA ladder; lanes 2, 9, 10, and 13: phylogroup A; lanes 3–7: phylogroup B1; lanes 8 and 11: phylogroup B2; lane 1: phylogroup D.

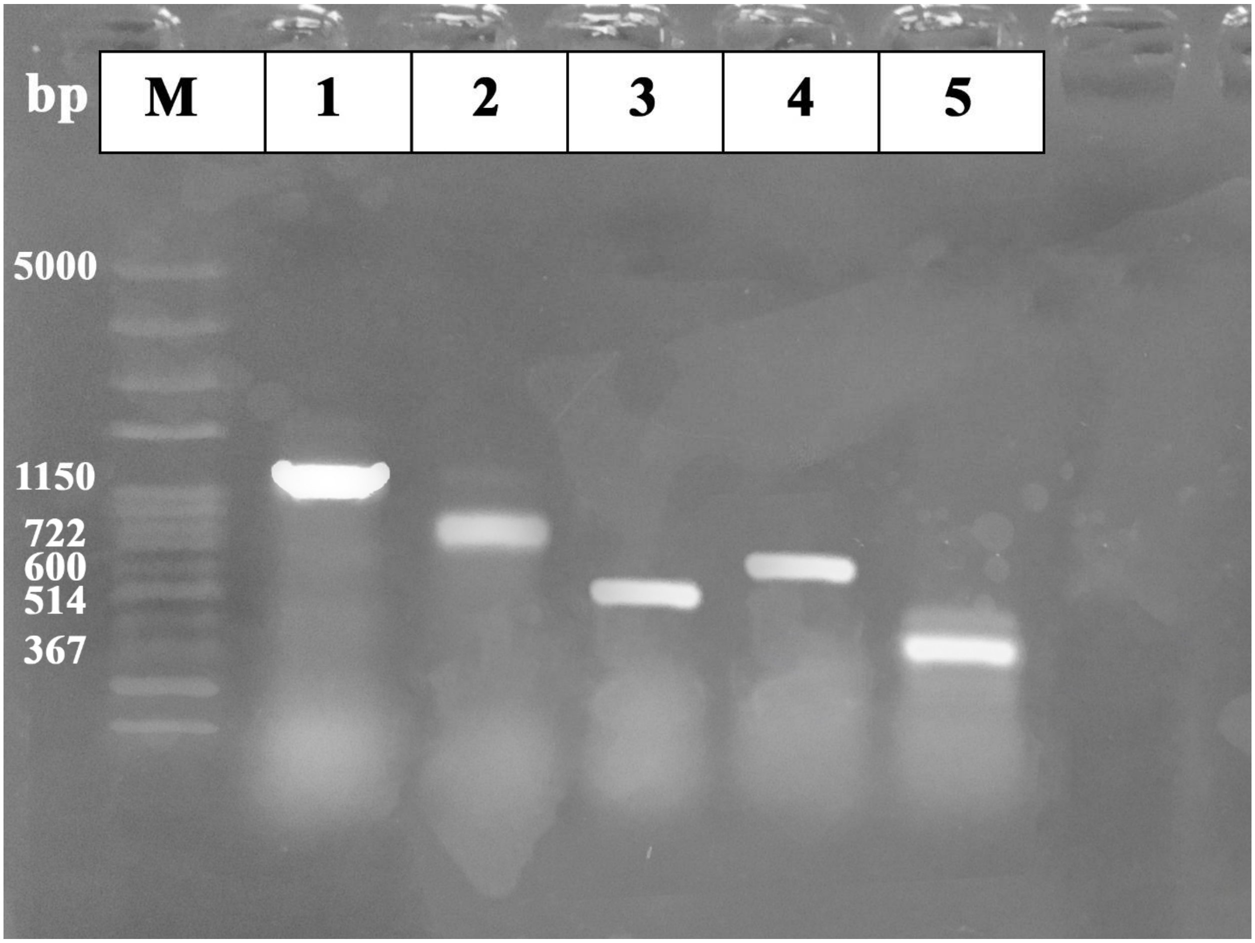

Figure S3: Agarose gel electrophoresis showing amplification of E.coli resistant genes: lane M: 100 bp DNA ladder; lane 1: blaTEM at 1150 bp; lane 2: sul2 at 722 bp; lane 3: aphA1 at 600 bp; lane 4: qnrA at 514 bp; lane 5: dfrA1 at 367 bp.

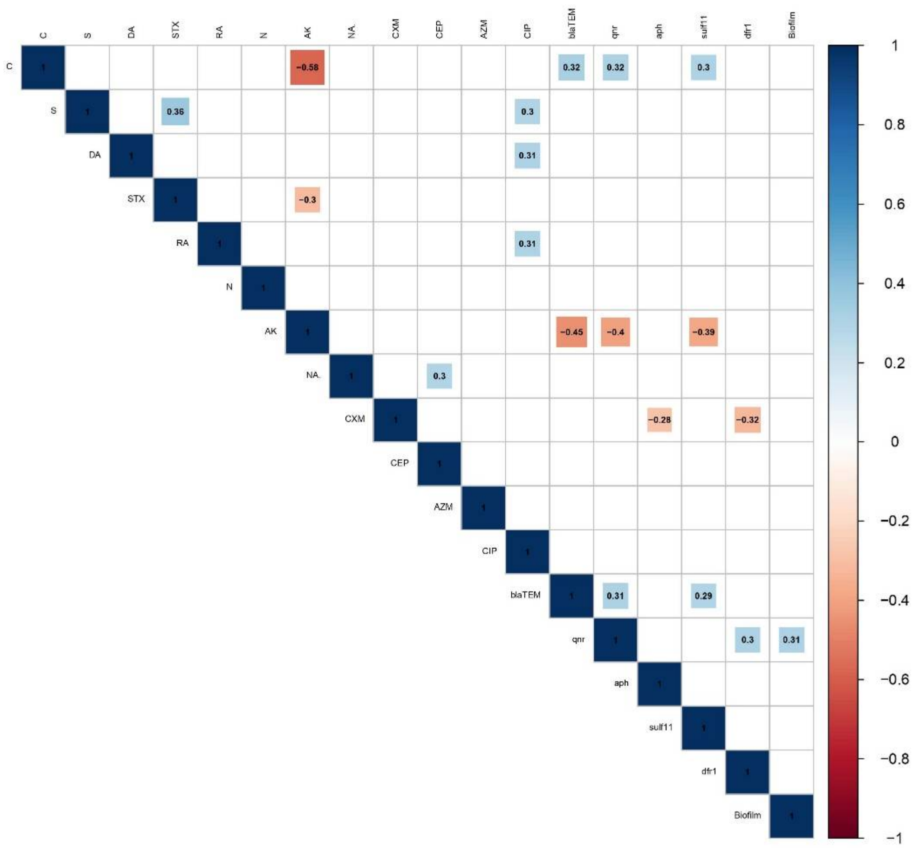

Figure S4: Association of antimicrobial resistance phenotypes, resistance genotypes, and biofilm production among the examined *E. coli* isolates. Blue and red boxes denote positive and negative correlations, respectively. Significant associations were calculated at $p < 0.05$; blank boxes denote non-significant correlations.
